# Clinical Spectrum and Functional Consequences Associated with Bi-Allelic Pathogenic *PNPT1* Variants

**DOI:** 10.3390/jcm8112020

**Published:** 2019-11-19

**Authors:** Rocio Rius, Nicole J. Van Bergen, Alison G. Compton, Lisa G. Riley, Maina P. Kava, Shanti Balasubramaniam, David J. Amor, Miriam Fanjul-Fernandez, Mark J. Cowley, Michael C. Fahey, Mary K. Koenig, Gregory M. Enns, Simon Sadedin, Meredith J. Wilson, Tiong Y. Tan, David R. Thorburn, John Christodoulou

**Affiliations:** 1 Murdoch Children’s Research Institute, Melbourne, VIC 3052, Australia; 2 Department of Paediatrics, University of Melbourne, Melbourne, VIC 3052, Australia; 3 Kids Research, The Children’s Hospital at Westmead, Sydney, NSW 2145, Australia; 4 Discipline of Child & Adolescent Health, Sydney Medical School, University of Sydney, Sydney, NSW 2050, Australia; 5 Department of Neurology, Perth Children’s Hospital, Perth, WA 6009, Australia; 6 Department of Metabolic Medicine and Rheumatology, Perth Children’s Hospital, Perth, WA 6009, Australia; 7 Genetic Metabolic Disorders Service, Western Sydney Genetics Program, The Children’s Hospital at Westmead, Sydney, NSW 2145, Australia; 8 Discipline of Genetic Medicine, Sydney Medical School, University of Sydney, Sydney, NSW 2145, Australia; 9 Victorian Clinical Genetic Services, Melbourne, VIC 3052, Australia; 10 Precision Medicine Theme, Children’s Cancer Institute, Kensington, NSW 2750, Australia; 11 Kinghorn Centre for Clinical Genomics, Garvan Institute, University of New South Wales, Randwick, NSW 2010, Australia; 12 School of Women’s and Children’s Health, University of New South Wales, Randwick, NSW 2031, Australia; 13 Department of Paediatrics, Monash University, Melbourne, VIC 3168, Australia; 14 The University of Texas McGovern Medical School, Houston, TX 77030, USA; 15 Department of Pediatrics, Division of Medical Genetics, Stanford University, Stanford, CA 94305, USA; 16 Department of Clinical Genetics, The Children’s Hospital at Westmead, Sydney, NSW 2145, Australia; 17 Discipline of Genomic Medicine, Faculty of Medicine and Health, University of Sydney, Sydney, NSW 2006, Australia

**Keywords:** mitochondrial, *PNPT1*, PNPase, interferon, OXPHOS, respiratory chain, mutation, splice defect

## Abstract

*PNPT1* (PNPase—polynucleotide phosphorylase) is involved in multiple RNA processing functions in the mitochondria. Bi-allelic pathogenic *PNPT1* variants cause heterogeneous clinical phenotypes affecting multiple organs without any established genotype–phenotype correlations. Defects in PNPase can cause variable combined respiratory chain complex defects. Recently, it has been suggested that PNPase can lead to activation of an innate immune response. To better understand the clinical and molecular spectrum of patients with bi-allelic *PNPT1* variants, we captured detailed clinical and molecular phenotypes of all 17 patients reported in the literature, plus seven new patients, including a 78-year-old male with the longest reported survival. A functional follow-up of genomic sequencing by cDNA studies confirmed a splicing defect in a novel, apparently synonymous, variant. Patient fibroblasts showed an accumulation of mitochondrial unprocessed *PNPT1* transcripts, while blood showed an increased interferon response. Our findings suggest that functional analyses of the RNA processing function of PNPase are more sensitive than testing downstream defects in oxidative phosphorylation (OXPHPOS) enzyme activities. This research extends our knowledge of the clinical and functional consequences of bi-allelic pathogenic *PNPT1* variants that may guide management and further efforts into understanding the pathophysiological mechanisms for therapeutic development.

## 1. Introduction

*PNPT1* encodes for polynucleotide phosphorylase (PNPase), a conserved homotrimeric 3′-to-5′ exoribonuclease predominantly localized in the mitochondrial matrix and intermembrane space [1]. It is primarily involved in mitochondrial RNA (mtRNA) processing and degradation [2]. 

PNPase has been suggested to play a role in RNA import into mitochondria [3,4]; however, experimental data have been contradictory and, to date, there is no general agreement about an RNA import mechanism [5]. Recent reports suggest that disrupted PNPase RNA processing could lead to the accumulation of double-stranded mtRNAs, with the possibility of triggering an altered immune response [6,7].

Patients with bi-allelic *PNPT1* pathogenic variants have shown wide clinical heterogeneity ranging from non-syndromic hearing loss to multisystemic Leigh syndrome [8,9]. To date, no clear phenotype–genotype correlations have been drawn. 

In a bid to better understand the clinical phenotype and functional consequences of patients with *PNPT1*-related diseases, we reported seven new patients with bi-allelic *PNPT1* variants and expanded upon the mutational spectrum. We also conducted functional studies and performed a thorough clinical review of previously published patients with *PNPT1* variants [6,8,9,10,11,12,13]. 

## 2. Experimental Section

### 2.1. Patients

We report seven new patients (P1, 2, 3, 3.2, 4, 7, 8) with bi-allelic variants in *PNPT1*, and provided updates for three previously published cases (P5, 6, 9). We also conducted a PubMed search using the terms PNPase (All Fields) OR (PNPT1 (All Fields) AND (“persons” (MeSH Terms) OR “persons” (All Fields) OR “individual” (All Fields))) up to September 2019 for publications limited to human subjects to describe the clinical features of all the patients with bi-allelic *PNPT1* pathogenic variants reported in the literature to date [6,8,9,10,11,12,13]. 

Functional studies were conducted in samples collected from four patients (P1, 2, 3, 4) in which the *PNPT1* variants were identified by whole genome sequencing (WGS; Garvan Institute, Sydney) or whole exome sequencing (WES; Victorian Clinical Genetics Services (VCGS), Melbourne; Broad Institute, Cambridge, MA, USA; and Baylor College of Medicine, Houston, TX, USA). 

This study was performed in accordance with the Helsinki Declaration and ethical standards of the responsible ethics committees. The project was approved by the Human Research Ethics Committees of the Sydney Children’s Hospitals Network (ID number HREC/10/CHW/114), Melbourne Health (ID number HREC/16/MH/251), and the Royal Children’s Hospital (ID number HREC/16/RCHM/150).

### 2.2. Next Generation Sequencing (NGS) and in Silico Tools

The variants in P1 were identified through whole exome sequencing (WES) performed by the Genomics Platform at the Broad Institute of Harvard and MIT (Broad Institute, Cambridge, MA, USA). The variants in P2 were identified through trio whole genome sequencing (WGS) performed at the Kinghorn Centre for Clinical Genomics (Garvan Institute, Sydney) as previously described [14]. The variants in P3 and P4 were identified through WES performed at Victorian Clinical Genetics Services (VCGS), Melbourne. P7 and P8 variants were identified through WES performed at Baylor College of Medicine, Medical Genetics Laboratories, Whole Genome Laboratory (Houston, TX, USA).

In silico prediction analyses were performed using PolyPhen-2 [15], SIFT [16], Combined Annotation Dependent Depletion CADD [17], MutationTaster [18], and Human Splicing Finder v3.1 [19]. Visualization of variants in the Pfam [20] protein domains was conducted with MutationMapper [21]. Allele frequencies were determined using the Genome Aggregation Database [22].

### 2.3. Western Blotting

Fibroblast protein extraction and Western blotting were performed using total Abcam OXPHOS human WB antibody cocktail (ab11041) and PNPase (ab96176) as previously published [10]. 

### 2.4. Mitochondrial Oxidative Phosphorylation (OXPHOS) Enzyme Activities

Spectrophotometric analysis of OXPHOS enzyme activities in muscle and fibroblasts was performed as previously described [23]. The mitochondrial respiratory chain complex I (CI) and complex IV (CIV) dipstick activity assays (Abcam, Melbourne, VIC, Australia) were performed using 15 μg of whole-cell lysates fibroblasts as previously published [10]. 

### 2.5. Fibroblast Culture, RNA Extraction, and Complementary DNA (cDNA) Studies

Cycloheximide treatment of cultured fibroblasts from P2 and controls was performed as published [24]. Cultured fibroblasts from patients P1, P2, P3, and P4, and three control lines were incubated in the presence or absence of 100 U/mL interferon α-2a Roferon-A (Roche, Sydney, Australia) in HyClone Dulbecco’s Modified Eagle Medium (GE Healthcare, Rydalmere, NSW, Australia) at 37 °C and 5% CO_2_ for 24 h.

RNA was isolated from fibroblasts using the RNeasy Plus kit (Qiagen, Hilden, Germany) following the manufacturer’s instructions. Reverse transcription was performed using SuperScript III First-Strand synthesis kit (Thermo Fisher Scientific, Carlsbad, CA, USA) following the manufacturer’s instructions. 

### 2.6. RNA Extraction from Blood

PAXgene blood RNA tubes (PreAnalytix by Qiagen, Hombrechtikon, Switzerland) were used to collect peripheral blood samples. After collection, the tubes were left at room temperature between 2 h and 72 h before extracting RNA using the PAXgene blood RNA kit (PreAnalytix by Qiagen) following the manufacturer’s instructions. 

### 2.7. PCR Quantification of Unprocessed Mitochondrial Transcripts

qPCR for the quantification of unprocessed transcripts was performed with AccuPower 2X Greenstar qPCR Master Mix (Bioneer, Daejeon, Korea) using primers previously published by Matilainen and collaborators [9]. The relative accumulation of unprocessed mitochondrial transcripts was calculated using the 2(-ΔΔCt) method [25].

### 2.8. Interferon Signature Analysis

The relative expression of six interferon-stimulated genes was analyzed by qPCR using cDNA from 40 ng RNA, TaqMan Fast Advanced Master Mix (Thermo Fisher Scientific), and Taq Man probes for *IFI27* (Hs01086370_m1), *IFI44L* (Hs00199115_m1), *IFIT1* (Hs00356631_g1), *ISG15* (Hs00192713_m1), *RSAD2* (Hs01057264_m1), and *SIGLEC1* (Hs00988063_m1). The relative abundance was normalized to *HPRT1* (Hs03929096_g1) and *18S* (Hs999999001_s1). Median expression was used to calculate an interferon score as described by Dhir, Rice, and collaborators [6,26].

## 3. Results

**Figure 1:**
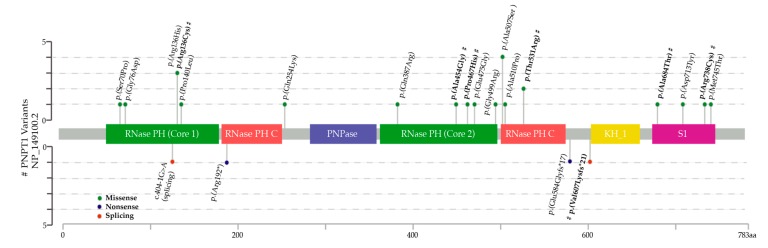
Lollipop plot depicting the polynucleotide phosphorylase (PNPase) protein and location of pathogenic variants reported in 30 alleles (15 families). Missense variants are shown above the protein. Nonsense and splicing variants are shown below. Novel variants reported in this article are marked with #.

A total of 24 patients with bi-allelic variants in *PNPT1* were identified in 15 families, including seven new patients (P1, 2, 3, 3.1, 4, 7, 8), three previously published individuals with additional clinical information since the previous publication (P5, 6, 9), and fourteen other patients reported in the literature (Tables S1 and S2). A total of 22 different pathogenic variants were found, of which only three were reported in more than one family. The p.(Ala507Ser) variant was reported in 27% of the families (4/15), followed by the p.(Thr531Arg) variant at 13% (2/15), and p.(Arg136His) at 13% (2/15) (Figure 1). 

Most of the patients (75% *n* = 18) presented within the first year of life. The most common initial manifestations were tone abnormalities (42% *n* = 10), feeding difficulties (17% *n* = 4), and mild to severe sensorineural hearing loss (38% *n* = 9). Other reported initial features were regression, choreoathetosis, visual loss, and cataracts (Table S1).

The median age at the last follow up was 8.9 years (range 1–78). Three of the patients died due to acute encephalopathy (P15 at 4 years), infection (P16 at 2.4 years), and unstated reasons (P21 at 2 years) (Table S1).

**Figure 2:**
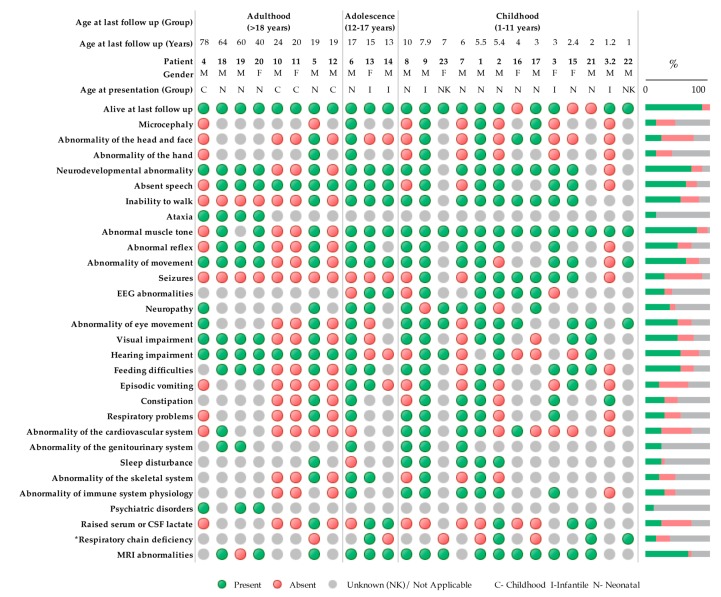
Clinical symptoms using Human Phenotype Ontology (HPO) terminology of patients with bi-allelic *PNPT1* variants (*n* = 24). Green indicates a present phenotype, red indicates an absent phenotype, and gray is unknown or not applicable. * Respiratory chain deficiencies refer to reported reductions in oxidative phosphorylation (OXPHOS) enzyme activity measured by spectrophotometric analysis in any tissue. Neonatal (0–28 days), infant (28 days–1 year), and childhood (>1 year).

The clinical symptoms of all the reported patients to date are summarized in Figure 2 and Table S1.

### 3.1. cDNA Studies Identify a Splicing Defect in P2

**Figure 3:**
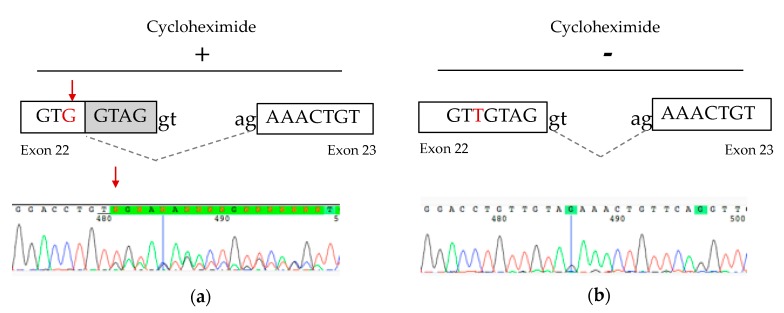
cDNA studies from P2 fibroblast cells. (**a**) Schematic representation and partial electropherogram showing the creation of a new splice site generated by the NM_033109.4:c.1818T>G variant in cells treated with cycloheximide. (**b**) Without cycloheximide treatment, the heterozygous NM_033109.4:c.1818T>G variant was not detectable, suggesting that the mutant RNA is subject to nonsense-mediated decay.

The NM_033109.4:c.1818T>G heterozygous variant identified in P2 was originally believed to be synonymous p.(Val606=), however it was located 5 bp from the donor splice site and in silico studies suggested the possibility of a splicing defect (Table S2). cDNA studies performed from cultured fibroblasts grown with and without cycloheximide treatment to inhibit nonsense-mediated decay (NMD) confirmed a splicing defect resulting in a frameshift deletion and premature termination codon p.(Val607Lysfs*21), leading the transcript to be subject to degradation by NMD (Figure 3).

### 3.2. Mitochondrial OXPHOS Protein Expression and Activity

**Figure 4:**
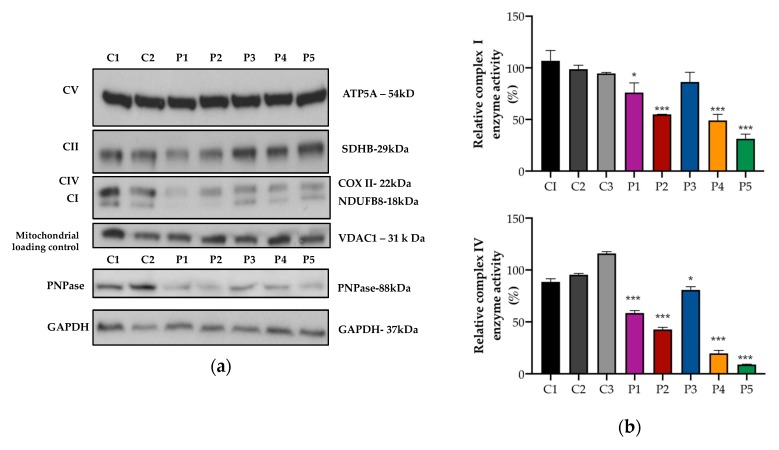
Protein expression and OXPHOS activity in fibroblasts. (**a**) Representative images of Western blot analysis performed in fibroblast proteins from controls (C) and patients (P). In the patients, there was a reduction in protein expression of PNPase (lower panel) and complex I and complex IV OXPHOS mitochondrial subunits (upper panel). (**b**) Complex I (upper panel) and complex IV (lower panel) enzyme activity was analyzed using dipstick activity assays. In the patients, there was a mild/moderate reduction in complex I and complex IV enzyme activities. The mean and variation (SEM) between three independent experiments are shown. (*** *p* < 0.001, * *p* < 0.033).

In the fibroblasts, there was a reduction of the mitochondrial complex IV COX II subunit and the complex I NDUFB8 subunit in patients P1–4, although the levels of other OXPHOS subunits were normal (Figure 4a). The activities of complex I and complex IV were determined using a dipstick assay. Compared to the control means, there was a relatively mild reduction in complex I by 20% (P1), 45% (P2), 50% (P4), and a modest reduction in complex IV by 40% (P1), 57% (P2), 20% (P3), and 80% (P4) (Figure 4b). P5 was a previously reported patient with *PNPT1* variants [10] and was used as a positive control.

However, spectrophotometric analysis of OXPHOS enzymes was normal in fibroblasts from P1, and was only borderline low for complex IV activity relative to protein and citrate synthase in muscle from P2 (Table 1).

### 3.3. Accumulation of Mitochondrial Unprocessed Transcripts

To assess the PNPase mitochondrial RNA processing activity, we measured the accumulation of unprocessed polycistronic mitochondrial transcripts in fibroblasts.

**Figure 5:**
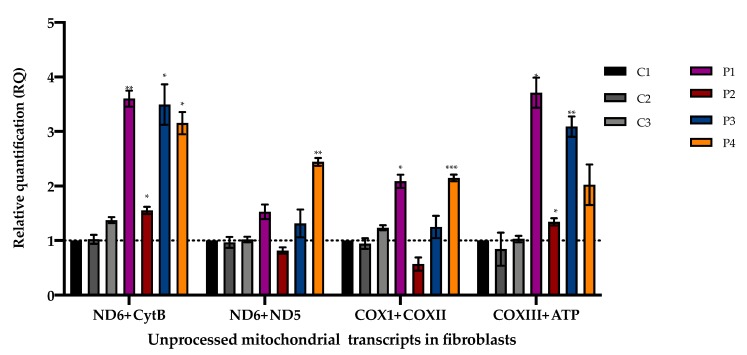
Unprocessed polycistronic mitochondrial transcripts in fibroblasts measured by qPCR. Mean and variation (SEM) between three independent experiments are shown (*** *p* < 0.001, ** *p* < 0.002, * *p* < 0.033).

Compared to controls, fibroblasts from patients P1–4 showed an accumulation of aberrantly processed mitochondrial RNA transcripts (Figure 5) in line with the PNPase function in mtRNA processing. The accumulation of unprocessed transcripts in myoblasts from a patient with bi-allelic pathogenic variants in *PNPT1* was previously described [9]. Notably, our patients presented different patterns of accumulation, which were not restricted to transcripts around *MT-ND6*. Further studies may help us to understand if the differences in the accumulation of unprocessed mitochondrial transcripts are related to specific *PNPT1* variants.

### 3.4. Interferon Signature in Patients with PNPT1 Variants

**Figure 6:**
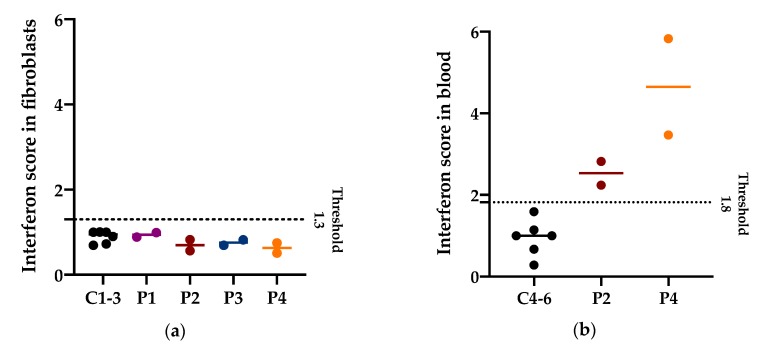
Interferon (IFN) score in fibroblasts and in blood. (**a**) Median and individual values of the IFN score in fibroblasts of three controls and patients with *PNPT1* variants (P1–4) from two independent experiments. The IFN score threshold of 1.3 corresponds to the mean IFN score of controls + 2SD. Higher values are considered positive. (**b**) Median and individual values of the IFN score in blood of three controls and patients with *PNPT1* variants (P2 and P4) from two independent experiments. The IFN score threshold of 1.8 corresponds to the mean IFN score of controls + 2SD. Higher values are considered positive.

Increased expression of interferon-stimulated genes has been previously described in blood from P14, P22, and P23 with bi-allelic *PNPT1* variants [6]. To assess if the PNPase defects could elicit an increased interferon response in different tissues, we calculated the interferon score in fibroblasts (P1–4) and in blood (only available from P2 and P4). In fibroblasts from P1–4 stimulated with IFN-α2a (100 U/mL), the interferon response was not different to controls (interferon score below the 1.3 threshold in fibroblasts). On the other hand, in blood from P1 and P4, a positive interferon score was identified with a median of 2.53 and 4.65, respectively (above the 1.8 threshold in blood) (Figure 6).

## 4. Discussion

This cohort illustrated the marked phenotypic heterogeneity associated with bi-allelic *PNPT1* variants. Severe developmental delay and regression were the most common neurodevelopmental abnormalities. Sensorineural hearing loss, optic atrophy, tone, and movement abnormalities—including choreoathetosis, dystonia, myoclonus, and ataxia—were also amongst the most prevalent clinical features. Most patients had a history of hypotonia (*n* = 19) and four patients presented a pattern of central hypotonia with peripheral hypertonia. Brain imaging was available in 17 patients, of which 16 patients had MRI abnormalities. The most common were white matter abnormalities (*n* = 8), followed by thin corpus callosum (*n* = 5), and basal ganglia abnormalities (*n* = 4) (Figure S1).

Interestingly, most of the patients in the cohort had normal to mildly deficient OXPHOS when measured by spectrophotometric enzyme assays. This finding has several implications. First, a normal OXPHOS enzyme analysis does not rule out the diagnosis of *PNPT1*-related disease. Second, reduced OXPHOS protein expression or activity measured by dipstick assays does not always correlate with spectrophotometric assays, as shown in P1 and P2. This discrepancy could be attributed to sample preparation and differences in the methodology, which has been discussed previously [10]. Finally, in order to confirm pathogenicity, functional analyses focusing on the primary role of *PNPT1* (mtRNA processing) and interferon signaling in blood rather than fibroblasts appear to be more sensitive, and potentially more specific than evaluating the downstream effects on the OXPHOS enzyme activities.

Most of the patients with *PNPT1*-related disease have been identified through NGS approaches. The identification of an apparently synonymous variant in P2 that was later demonstrated to result in abnormal mRNA splicing highlights the importance of careful interpretation of apparently “silent” variants in NGS data, the utility of in silico tools, and the need for cDNA studies when following up potential splicing variants.

The identification of an interferon signature in blood from patients with *PNPT1* variants raises the possibility that an immune response could contribute to disease pathogenesis [6]. Further longitudinal studies could elucidate if there is a correlation between the interferon signature and disease severity, or the possibility of being a useful clinical biomarker and/or therapeutic target.

So far, little is known about possible genotype–phenotype correlations, and it is not clear what factors could be contributing to the clinical spectrum of *PNPT1*-related diseases. Studies suggest that the rate of disease progression could be, in part, related to having two deleterious variants affecting the first core domain [9]. In line with this, three patients with rapidly progressive disease and early death (median age 2.4 years) were described (Table S1), in which the variants were located in the first core domain. P21 had the p.(Arg136His) variant in a homozygous state. P15 also had the p.(Arg136His) variant but in compound heterozygosity with the p.(Pro140Leu) variant, which was also located in the first core domain. P16, who had a p.(Gly76Asp) located in the first core domain and a p.(Arg192*), was predicted to result in nonsense-mediated decay [6,9,12]. The novel variant p.(Arg136Cys) identified in P3 was an alternate amino acid change located in the same active site as described in P21 and P15, but her second pathogenic variant p.(Pro467His) was located in the second core domain. Interestingly, she is currently three years old and has a relatively slower progression. Her brother (P3.2) also seemed to be more mildly affected. Intrafamilial variability was also described in other families with *PNPT1* variants (P5, 6, 7, 8; Table S1), but bona fide genetic modifiers of disease severity have yet to be identified.

P4 had the longest survival reported to date (78 years), with a slowly progressive clinical course. He presented at 11 years old with hearing and visual loss. Subsequently, he developed blepharospasm and ptosis. Over the decades, he developed progressive ataxia, poor night vision, and external ophthalmoplegia. His hearing worsened during his 60s and, therefore, he received a cochlear implant. Eaton and collaborators [13] also described one family that presented with a slowly progressive pattern. Both affected individuals had congenital sensorineural hearing loss and developed neurological symptoms in adulthood. It is interesting to note that the pathogenic *PNPT1* variants in both these patients were distal variants located within the S1 domain. Further work and more patients are required to formally establish genotype–phenotype correlations.

Careful phenotypic analysis in rare monogenic diseases is valuable for identifying common clinical features [27,28]. In our cohort, this approach led us to describe the most common phenotypic findings associated with *PNPT1*-related disease that should increase the diagnostic suspicion of this genetic etiology, and could guide screening and management in patients with a molecular diagnosis. On the other hand, no single finding was present in 100% of the patients and, thus, the lack of any of these features does not rule out the diagnosis. Some of the clinical features have only been described in adult patients, such as obsessive–compulsive disorder (P19), depression (P4, P19), and paranoia (P20), or in single cases, such as renal artery stenosis in P9. The causal relationship of some of these findings is unknown and it remains to be seen whether some of these features are coincidental rather than linked to the pathophysiology of *PNPT1* dysfunction.

The diverse clinical heterogeneity of patients with bi-allelic *PNPT1* variants presents a diagnostic and management challenge to the clinician. As more patients are diagnosed, it is important to share detailed clinical information to understand the natural history, elucidate new insights into the mechanisms underlying the pathology, and aid with the identification of potential treatment strategies.

## Figures and Tables

**Table 1:** Spectrophotometric analysis of oxidative phosphorylation (OXPHOS) enzyme activities in fibroblasts from P1 and muscle from P2.

	P1 (Fibroblasts)			P2 (Muscle)		
Enzyme	Residual Activity (%)	CS Ratio (%)	CII Ratio (%)	Residual Activity (%)	CS Ratio (%)	CII Ratio (%)
I	150	107	75	75	73	126
II	194	140	-	59	58	-
III	266	187	133	69	65	113
IV	91	67	47	34	34	59
CS	137			102		

Activities of OXPHOS enzyme complexes I, II, III, IV, and citrate synthase (CS) are expressed as % relative to protein (residual activity), citrate synthase (CS ratio), and CII (CII ratio) of control samples.

## Supplementary Materials

The following are available online at https://www.mdpi.com/2077-0383/8/11/2020/s1, Table S1: Clinical phenotype of patients with bi-allelic PNPT1 variants; Table S2: In silico studies and population frequency of PNPT1 variants identified in new families; Figure S1: Brain MRI findings in patients with bi-allelic *PNPT1* variants.
