# Supplementary material for: Clinical Spectrum and Functional Consequences Associated with Bi-Allelic Pathogenic PNPT1 Variants
Source: J Clin Med. 2019 Nov 19;8(11):2020. doi: 10.3390/jcm8112020 (PMC6912252; doi:10.3390/jcm8112020)
Supplement: Supplementary file 1 [file jcm-08-02020-s001.zip › supplementary files/Figure S1.docx]

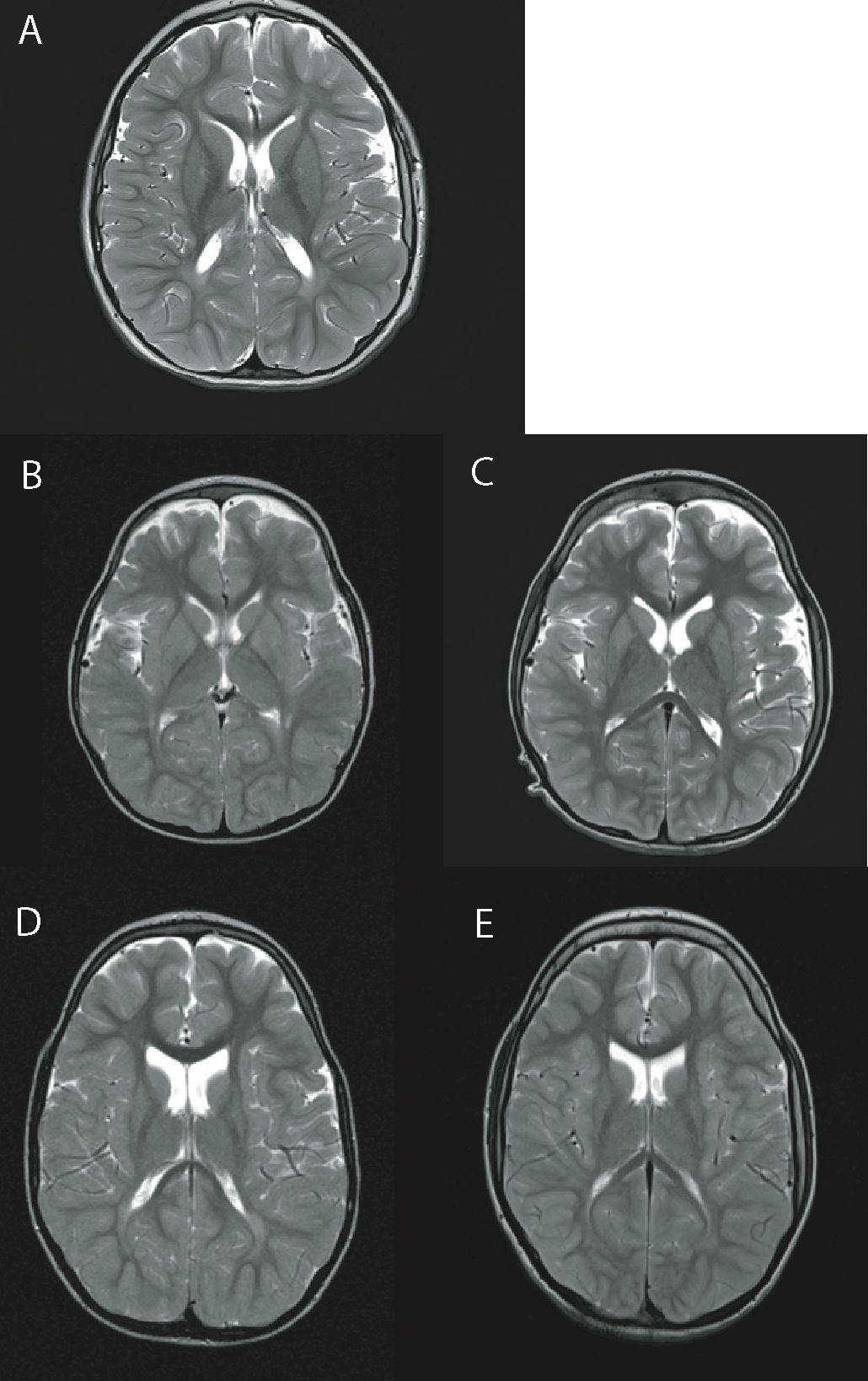


**Figure S1.** Brain MRI findings in patients with bi-allelic *PNPT1* variants, T2-weighted axial view showing **(a)** generalized neuroparenchymal volume loss with a posterior predilection in patient 2 at 5 years (**b**) mild white matter atrophy in patient 5 at age 3 years and **(c)** at 11 years, **(d)** decrease in white matter, non-specific white matter changes in the periventricular and parieto-occipital regions in patient 6 at 5 years and **(e)** at 13 years.
